# Systematic CpT (ApG) Depletion and CpG Excess Are Unique Genomic Signatures of Large DNA Viruses Infecting Invertebrates

**DOI:** 10.1371/journal.pone.0111793

**Published:** 2014-11-04

**Authors:** Mohita Upadhyay, Neha Sharma, Perumal Vivekanandan

**Affiliations:** Kusuma School of Biological Sciences, Indian Institute of Technology Delhi, New Delhi, India; University of Bonn, Institute of Experimental Haematology and Transfusion Medicine, Germany

## Abstract

Differences in the relative abundance of dinucleotides, if any may provide important clues on host-driven evolution of viruses. We studied dinucleotide frequencies of large DNA viruses infecting vertebrates (n = 105; viruses infecting mammals = 99; viruses infecting aves = 6; viruses infecting reptiles = 1) and invertebrates (n = 88; viruses infecting insects = 84; viruses infecting crustaceans = 4). We have identified systematic depletion of CpT(ApG) dinucleotides and over-representation of CpG dinucleotides as the unique genomic signature of large DNA viruses infecting invertebrates. Detailed investigation of this unique genomic signature suggests the existence of invertebrate host-induced pressures specifically targeting CpT(ApG) and CpG dinucleotides. The depletion of CpT dinucleotides among large DNA viruses infecting invertebrates is at least in part, explained by non-canonical DNA methylation by the infected host. Our findings highlight the role of invertebrate host-related factors in shaping virus evolution and they also provide the necessary framework for future studies on evolution, epigenetics and molecular biology of viruses infecting this group of hosts.

## Introduction

Differences in the relative abundance of dinucleotides provide interesting insights on virus evolution. CpG dinucleotides in particular have received a lot of attention. Depletion of CpG dinucleotides among viruses has been linked to selective mutational pressure [1], translational selection [2] and virus evolution [3]. Virus-related factors that contribute to virus evolution include the type of genetic material (DNA vs RNA and strandedness) and the genome size [4]. Host factors that contribute to virus evolution are poorly understood.

Mutational pressure at specific dinucleotides is a critical parameter for understanding evolution of viruses. CpG dinucleotides are heavily methylated (80%–90%) in vertebrate host genomes as opposed to low levels of methylation in invertebrate host genomes [5]–[7]. As a result vertebrate host genomes are more CpG depleted than are invertebrate host genomes [8], [9]. Among DNA viruses infecting vertebrates, most small DNA viruses (<10 kb) are CpG depleted [10], while medium- and large-DNA viruses show marginal depletion or near-normal levels of the expected CpG dinucleotide frequencies [11]. The most widely accepted explanations for depletion of CpG dinucleotides include (a) spontaneous deamination of 5-methylcytosine (within a CpG dinucleotide) leading to an irreversible C to T transition [8], [12], [13] and (b) avoidance of toll-like receptor 9-mediated immune response [14]. There are no studies investigating the dinucleotide frequencies among large DNA viruses infecting invertebrate hosts. In addition, several complete genome sequences of large DNA viruses have became available in the last decade, allowing systematic analysis of dinucleotide frequencies in this group of viruses. We believe that understanding the differences in dinucleotide biases, if any among large DNA viruses infecting vertebrate and invertebrate hosts may provide clues on virus evolution. Interestingly, host-driven variation in dinucleotide content of viral genomes has received much attention recently. We have recently demonstrated a link between host methylation capabilities and virus evolution based on the relative abundance of dinucleotide frequencies [3].

Codon usage bias is an important determinant of virus evolution. Both mutational pressure and translational selection may contribute to codon usage bias [11], [15], [16]. Codon usage bias has not been investigated among large DNA viruses infecting invertebrate hosts.

In this study, we investigate the differences in the relative abundance of dinucleotides, mutational pressure and codon usage bias between large DNA viruses infecting vertebrate- and invertebrate hosts. Well documented differences between the two host groups include (a) Depletion of CpG dinucleotides in the vertebrate host genomes [7] (b) Higher rates of non-canonical DNA methylation (methylation of cytosines other than those within CpG dinucleotides) among invertebrate hosts [17], [18] (c) TLR 9-mediated selection pressure in vertebrate hosts (absent in invertebrate hosts) [19]. Keeping in mind the differences between the two host groups and the fact that viruses often co-evolve with their hosts [3], [16], we hypothesize that there will be significant differences in the relative abundance of dinucleotides and codon usage bias between the large DNA viruses infecting vertebrate hosts and those infecting invertebrate hosts. We believe that the study will help identify host-specific constraints principally responsible for driving the evolution of large DNA viruses within a given host group.

## Materials and Methods

### Retrieval of DNA sequences

The available full-length sequences of large double-stranded DNA (ds-DNA) viruses infecting vertebrates and invertebrates were retrieved from NCBI virus genome resources (http://www.ncbi.nlm.nih.gov/genomes/GenomesGroup.cgi?taxid=35237) or http://www.ncbi.nlm.nih.gov/nucleotide). When multiple full-length sequences were available for a virus, only one full-length sequence was used for analysis. The genomes with annotated tRNAs were excluded from analysis. A total of 193 sequences were used in this study; this includes 88 sequences of large DNA viruses infecting invertebrates (host details: insects = 84; crustaceans = 4) and 105 sequences of large DNA viruses infecting vertebrates (host details: mammals = 99 aves = 6; reptiles = 1) (the accession numbers and names of all viruses along with their respective hosts are listed in Dataset S1). Large DNA viruses have a genome size of about 100 kb or longer [11]. Despite being biologically similar to large DNA viruses, viruses belonging to the family *Adenoviridae* were excluded from the study owing to their small genome size (28–45 kb) as compared to the large DNA viruses included in the study (average genome size 164 kb). In addition, viruses belonging to the family *Iridoviridae* and *Polydnaviridae* were also excluded from the study. The genomes of iridoviruses are known to encode DNA methyltransferases leading to heavy methylation of the cytosine residues of CpG dinucleotides [20], [21]; this could potentially influence our study aimed at investigating host-related evolutionary forces. The viruses in the family *Polydnaviridae* are composed of multiple segments of DNA including wasp genes and wasp non-coding DNA [22]; hence this group of viruses were excluded from our study.

### Calculation of dinucleotide frequencies

The observed/expected ratios for the dinucleotide XpY [(O/E)_XpY_] are generally calculated using the observed frequency of the dinucleotide *f*(*XY*), the frequencies of the mononucleotides *f*(*X*) and *f*(*Y*) and the length of the genome *G.* In other words, 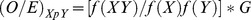. However, this calculation is suitable for organisms with single-stranded sequences [16]. In case of organisms with double-stranded sequences, opposite strand with the complementary nucleotides should also be considered while calculating the frequency of dinucleotides. In other words, in a double-stranded sequence, frequency of dinucleotide X_p_Y of one strand will be equal to the frequency of dinucleotide Y′_p_X′ in the complementary strand, where Y′ and X′ are complementary nucleotides to Y and X respectively.

Hence, the dinucleotide frequencies in a double-stranded sequence can be calculated using the following formula:
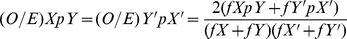
where, XpY denotes the dinucleotide in one strand, and Y′pX′ denotes the complementary dinucleotide in the opposite strand.

### Computation of codon usage frequencies

A freely available and widely used web tool, CodonW (http://bioweb.pasteur.fr/seqanal/interfaces/codonw.html) was used to determine the effective number of codon (ENC), total GC content and the nucleotide composition at the third codon position. The values of ENC range from 20–61, with a value of 20 representing maximum codon bias i.e one codon is used for one amino acid and a value of 61 represents no codon bias i.e. all the codons are equally used for each amino acid. ENC values of 35 or less are suggestive of significant codon usage bias.

The expected ENC value (ENC*) was calculated by using the following formula: 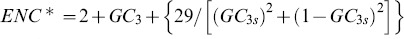 [23]. To determine how GC content influences codon usage, the relationship between ENC, ENC* and GC_3_ content was studied using an ENC-GC_3_ plot [23]. Another codon usage statistic, ENC′ was also calculated using the programs SeqCount and ENC prime [24]. ENC′ also ranges from 20–61 and is similar to ENC, except that ENC′ statistic corrects for the background nucleotide composition [24].

### Calculation of distribution of dinucleotides in the coding regions

The coding DNA sequences (CDS) as annotated in Genbank files were extracted using a web tool (http://www.cbs.dtu.dk/services/FeatureExtract/). The observed/expected ratios for the CDS (XpY _O/E*-*CDS_) were calculated.

### Neutrality plots

For each virus, total GC content and the frequency of nucleotides at the third (silent) codon position was calculated. In order to determine the relative effects of translational selection and mutation pressure, GC content at the third codon position (GC_3_) was plotted against the GC content at the first and second codon positions (GC_1,2_). The GC_1,2_ values were plotted against GC_3_ values in a scatter plot.

### Statistical analysis

Data were analyzed using Student’s *t* test, Wilcoxon signed-rank test and Pearson’s correlation coefficient (*r^2^*) as appropriate. Box plots, scatter plots and column (bar) graphs were made using MS-Excel or using the software Graph pad. On each box, the central horizontal line represents the median, the edges represent lower (Q1) and upper quartiles (Q3). Scatter plots were used to compare two parameters. Results were considered statistically significant at a *P* value of <0.05.

## Results

**Figure 1:**
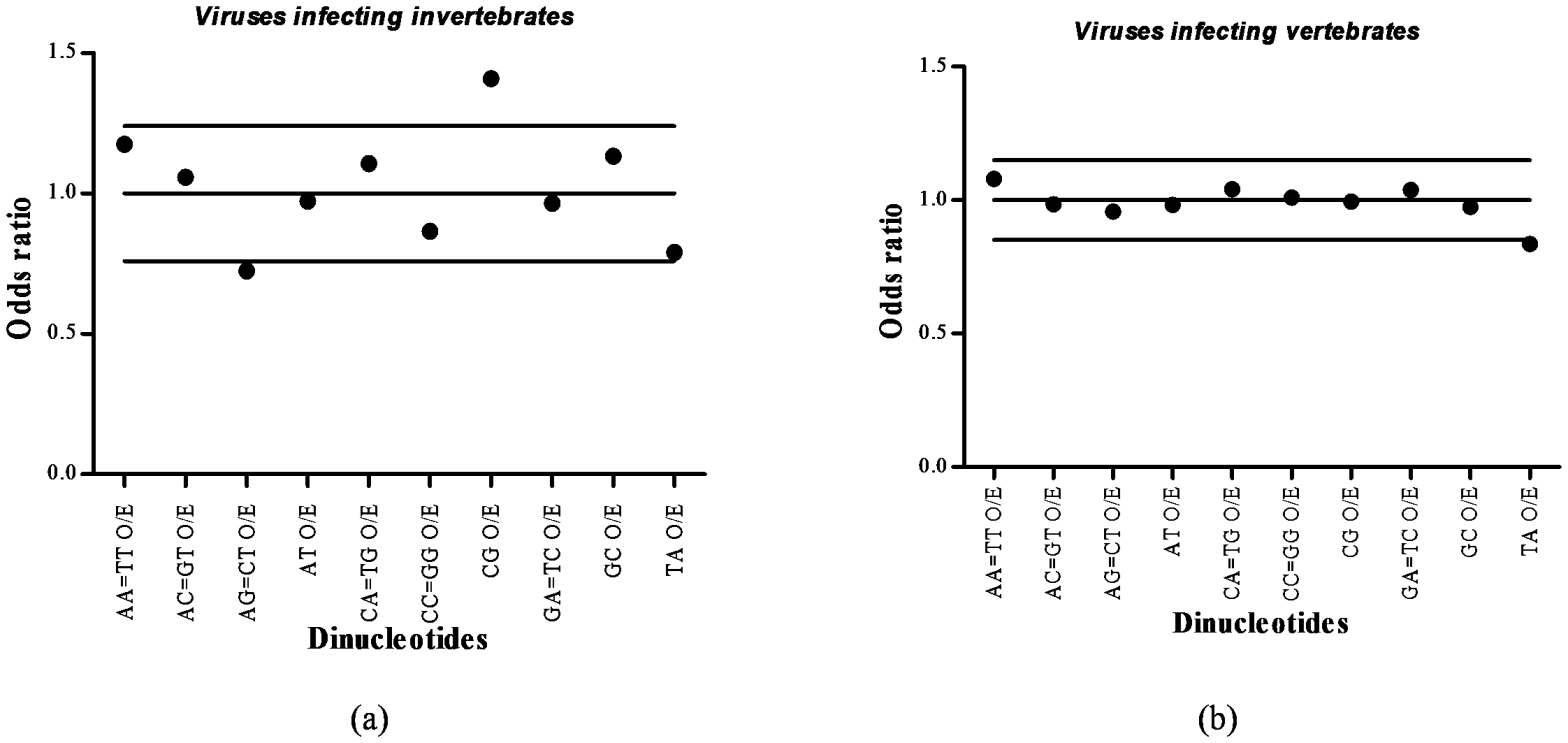
Dinucleotide usage patterns in large DNA viruses. (a) The mean±SD of dinucleotide O/E ratios (1.0±0.24) of large DNA viruses infecting invertebrates. For most dinucleotides the O/E ratios were located inside the confidence interval of 0.76–1.24 with the exception of CpT(ApG) dinucleotides (under-represented) and CpG dinucleotides (over-represented). (b) The mean±SD of dinucleotide O/E ratios (1.0±0.15) of large DNA viruses infecting vertebrates. The relative abundance of most dinucleotides was near-normal levels with the exception of TpA dinucleotide, which was under-represented.

The relative abundance of dinucleotides among large DNA viruses infecting invertebrates and vertebrates are summarized in Figure 1a and Figure 1b respectively. Since our study pertains to ds-DNA viruses, only 10 unique dinucleotides were used instead of 16 dinucleotides. For example, TT on the forward strand of a DNA sequence corresponds to AA on the reverse strand, so TT and AA were counted as one dinucleotide. For large DNA viruses infecting invertebrates the mean (±standard deviation (SD) value) dinucleotide O/E ratio is 1.0±0.24 (confidence interval of 0.76–1.24; Figure 1a). For large DNA viruses infecting vertebrates the mean (±standard deviation (SD) value) dinucleotide O/E ratio is 1.0±0.15 (confidence interval of 0.85–1.15; Figure 1b). The TpA dinucleotide is found to be universally under-represented in both the groups of viruses studied. No other major dinucleotide bias (O/E ratios outside the confidence interval) was seen among large DNA viruses infecting vertebrate hosts. In contrast, CpT(ApG) depletion (mean±standard deviation (SD): 0.72±0.10) and CpG excess (mean±standard deviation (SD): 1.41±0.29) emerged to be the two most striking dinucleotide biases among large DNA viruses infecting invertebrate hosts. The CpT(ApG) dinucleotide was the most depleted dinucleotide (CpT dinucleotide O/E ratios vs all other dinucleotide O/E ratios; *P*<0.0001; Wilcoxon signed rank test) and the CpG dinucleotide was the most overrepresented dinucleotide (CpG dinucleotide O/E ratios vs all other dinucleotide O/E ratios; *P*<0.0001; Wilcoxon signed rank test) among large DNA viruses infecting invertebrate hosts.

**Figure 2:**
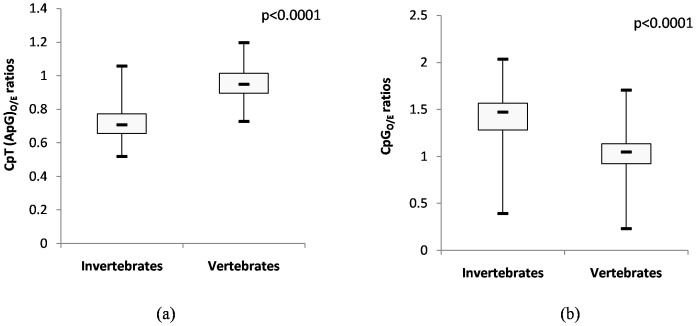
CpT(ApG) depletion and CpG excess among large DNA viruses infecting invertebrate hosts. (a) Box plot showing the distribution of CpT(ApG) dinucleotides among large DNA viruses infecting invertebrate- and vertebrate hosts. The depletion of CpT(ApG) dinucleotides is more pronounced among large DNA viruses infecting invertebrates as compared to those infecting vertebrates (mean±SD: 0.72±0.10 vs 0.96±0.09; *P*<0.0001). (b) Box plot showing the distribution of CpG dinucleotides among large DNA viruses infecting invertebrate- and vertebrate hosts. Large DNA viruses infecting invertebrates had a significantly higher CpG_O/E_ ratio than those infecting vertebrates (1.41±0.29 vs 0.99±0.26; *P*<0.0001).

The distribution of CpT(ApG) dinucleotides in large DNA viruses infecting invertebrates and vertebrates is shown in Figure 2a. Large DNA viruses infecting invertebrates had significantly lower CpT(ApG)_O/E_ ratios than those infecting vertebrates (mean±SD:0.72±0.10 vs 0.96±0.09; *P*<0.0001). Large DNA viruses infecting invertebrates had a significantly higher CpG_O/E_ ratios than those infecting vertebrates (1.41±0.29 vs 0.99±0.26; *P*<0.0001; Figure 2b). The distribution patterns of CpT and CpG dinucleotides are shown in Figure S1.

**Figure 3:**
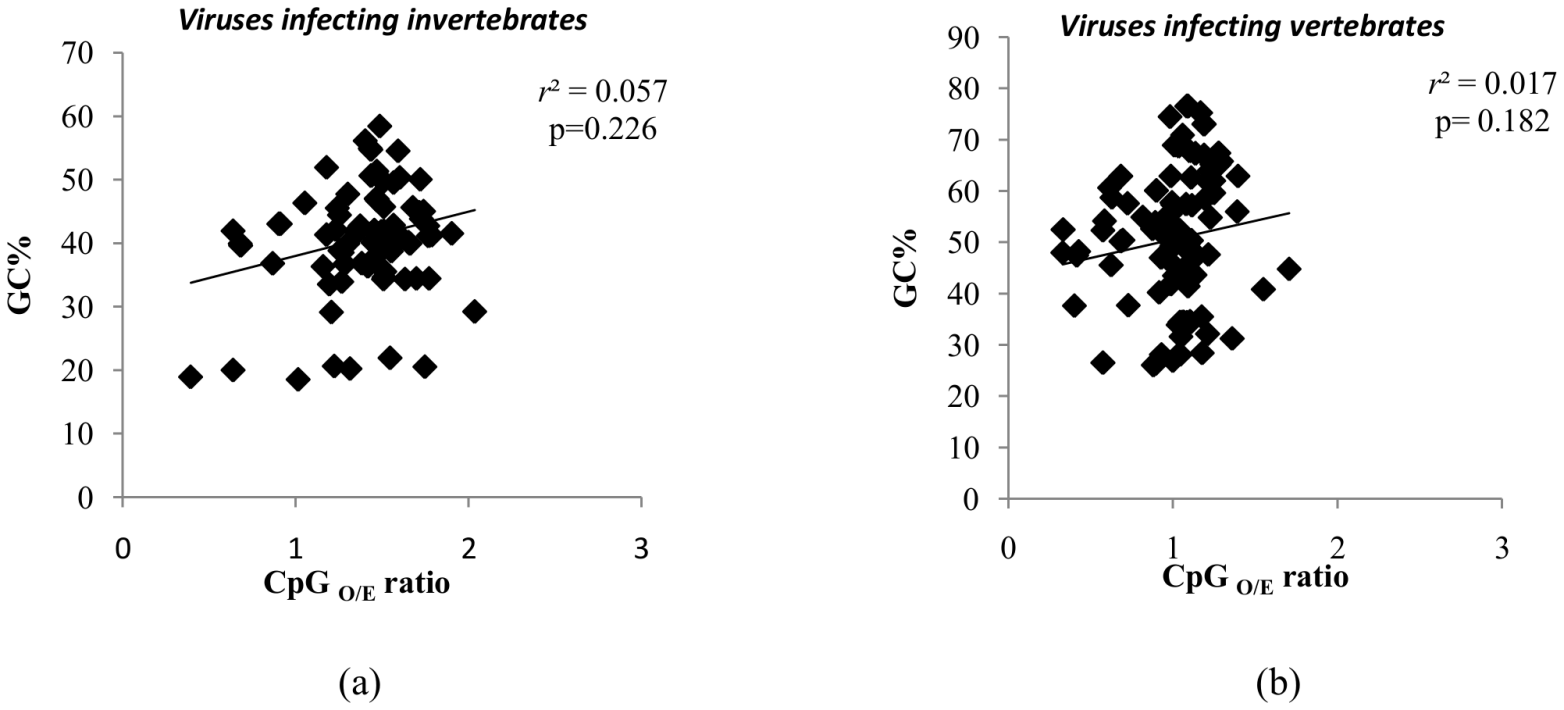
CpG_O/E_ ratios are not influenced by GC content. Scatter plot demonstrating the lack of correlation between CpG_O/E_ ratios (X-axis) and GC content (Y-axis) among (a) large DNA viruses infecting invertebrate hosts and (b) large DNA viruses infecting vertebrate hosts.

The GC content ranged from 19–58% in large DNA viruses infecting invertebrate hosts and between 26–77% in those infecting vertebrate hosts. A positive correlation between GC content and CpG dinucleotide frequencies has been demonstrated in previous studies [25], [26]. In our study, there was no correlation between CpG_O/E_ ratios and GC content (Figure 3a: *r^2^* = 0.057; *P* = 0.226 for the large DNA viruses infecting invertebrates; and Figure 3b: *r^2^* = 0.017; *P* = 0.182 for large DNA viruses infecting vertebrates).

**Figure 4:**
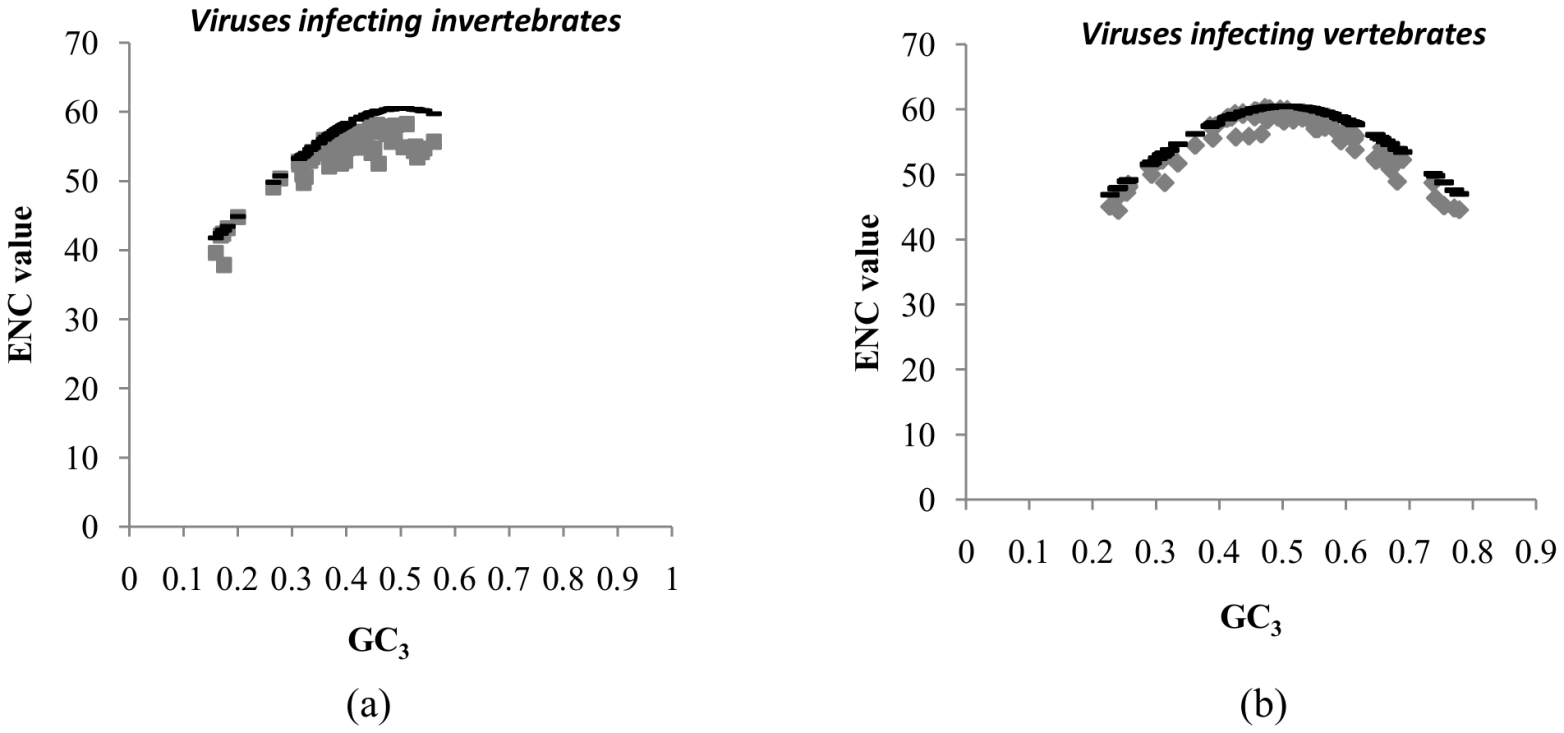
Lack of evidence for major codon usage biases. Correlation between GC content at third codon position (GC_3_) and the effective codon usage statistic (ENC) among (a) large DNA viruses infecting invertebrate hosts and (b) large DNA viruses infecting vertebrate hosts. The black line represents the expected ENC values (ENC*) calculated based on GC content. Most ENC values in both groups of viruses are on, or just below the ENC* values suggesting the absence of strong translational selection.

To investigate differences, if any in codon usage bias between the large DNA viruses infecting vertebrates and those infecting invertebrates we used the effective codon usage statistic, ENC (Effective number of codons) [11]. The ENC values ranged from 42.11 to 58.2 (mean±SD:53.77±4.02) for large DNA viruses infecting invertebrates and from 42.77 to 60.31 (mean±SD:54.83±4.58) for large DNA viruses infecting vertebrates. The ENC values clearly indicate the absence of major codon usage biases in both the groups of viruses. We examined the relationship between GC content at third codon position (GC_3_) and ENC values using ENC-GC_3_ plots. This relationship was then compared to the expected ENC value (ENC*) that would result if GC content primarily accounts for codon usage biases. In other words, ENC-GC_3_ plots will help assess the relative role of mutational pressure (ENC values lie on the expected ENC curve or just below the expected ENC curve) and translational selection (values would be considerably lower than the expected ENC curve). Interestingly, the actual values of ENC for both the groups of viruses lie on, or just below the expected ENC curve (Figure 4a and Figure 4b), indicating that codon usage bias is primarily explained by differences in GC composition and hence suggesting little or no role for translational selection.

**Figure 5:**
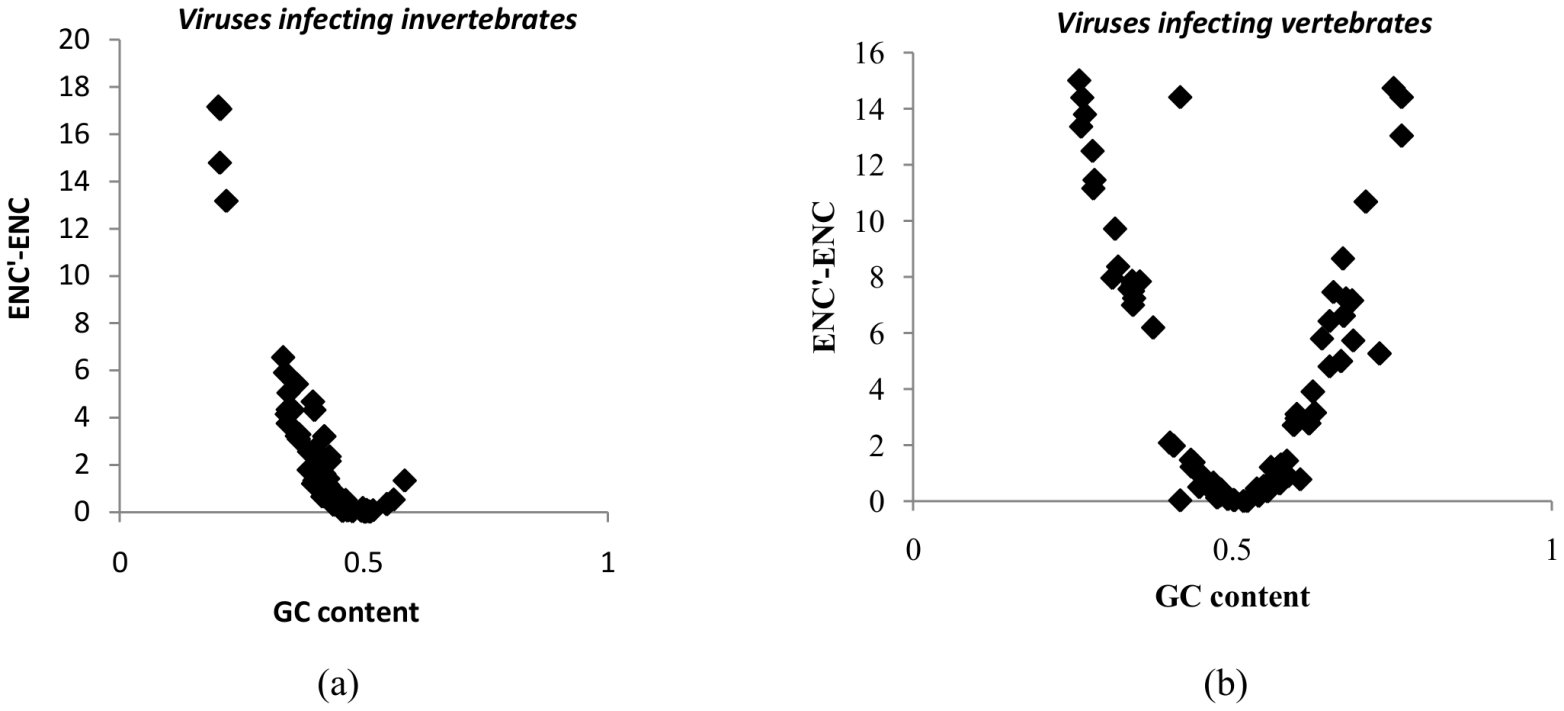
Observed differences in codon usage bias are primarily explained by the differences in the background nucleotide composition. Graphs showing the relationship between GC content and the difference between ENC′ and ENC (i.e. ENC′-ENC) among (a) large DNA viruses infecting invertebrate hosts and (b) large DNA viruses infecting vertebrate hosts. For most viruses (except for one virus), the ENC′ values were greater than ENC values; implying that the observed differences in codon usage bias are further reduced when corrected for background nucleotide composition.

The ENC statistic does not take into account the variation in nucleotide composition of the sequences studied [24]. ENC′ is a widely used statistic to measure codon usage bias and it takes into account the inherent differences in nucleotide composition of the sequence [24]. Higher the ENC′ values lower the codon usage bias. The ENC′ values ranged from 52.46 to 59.37 (mean±SD:56.74±1.68) for large DNA viruses infecting invertebrates and from 56.21 to 60.38 (mean±SD:59.23±0.93) for large DNA viruses infecting vertebrates. Notably, all ENC′ values (except for 1 virus) were higher than ENC values. This finding suggests that after correction for the observed background nucleotide composition there is no evidence of notable codon usage bias in either group of viruses studied. The difference between ENC′ values and ENC values (ENC′-ENC) are plotted against GC content in Figure 5a and 5b.

**Figure 6:**
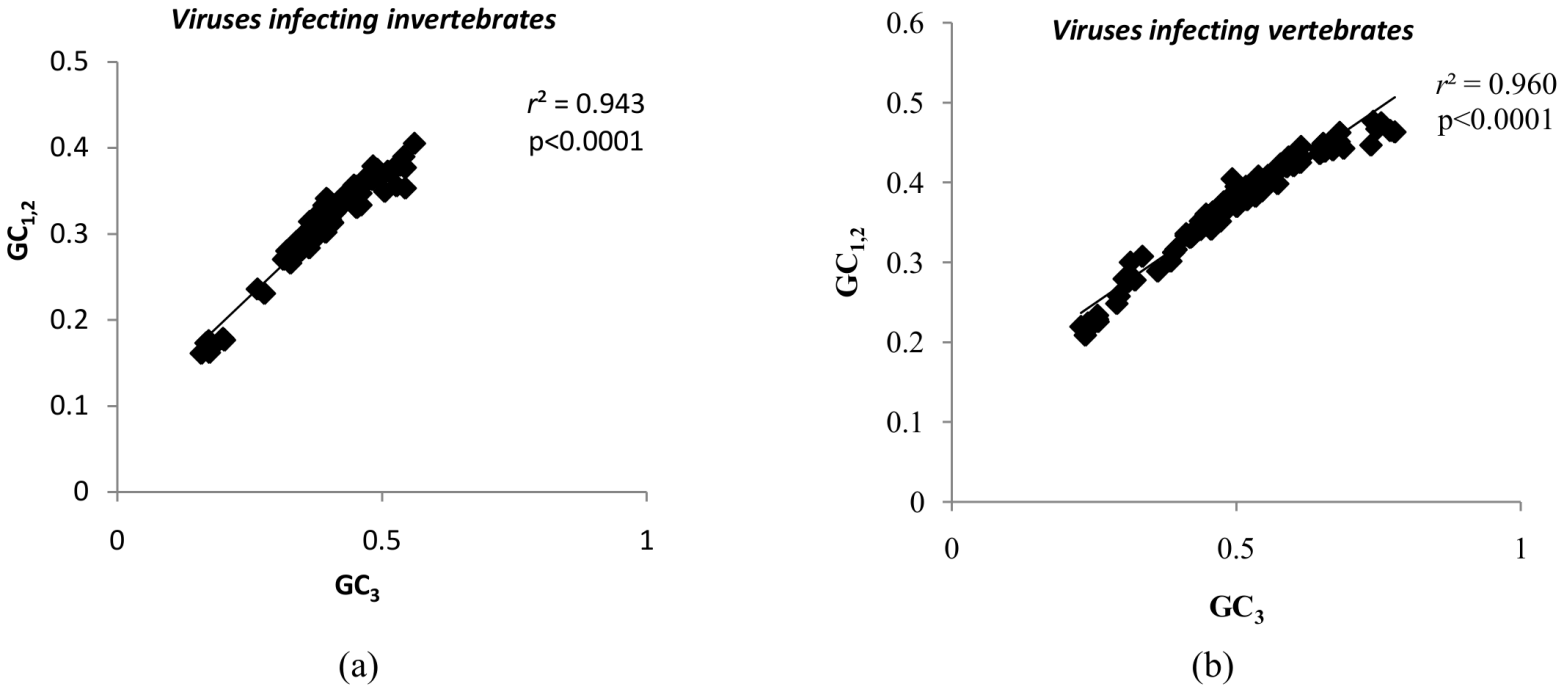
Neutrality plot. Evolution of large DNA viruses is primarily governed by mutational pressure. Scatter plot demonstrating a strong, near-perfect correlation between GC at the synonymous third codon position (GC_3_) (X-axis) and non-synonymous first/second codon positions (GC_1,2_) (Y-axis) among (a) large DNA viruses infecting invertebrate hosts and (b) large DNA viruses infecting vertebrate hosts. This finding suggesting that all codon positions are similarly affected and hence mutational pressure and not translational selection is primarily responsible for the observed differences in nucleotide composition among large DNA viruses.

Nucleotide composition among the three codon positions in both group of viruses was further examined by comparing the GC content at the synonymous third position (GC_3_) with GC content at non-synonymous first and second codon position (GC_1,2_) (Figure 6a and 6b). The correlation between GC_3_ and GC_1,2_ is often used to understand the role of mutational pressure and/or translational selection influencing nucleotide composition. In our study, we found significant correlation between GC_3_ and GC_1,2_ in both the groups of viruses (*r* ^2^ = 0.943 for large DNA viruses infecting invertebrates, *P*<0.0001, Figure 6a and *r* ^2^ = 0.960 for those infecting vertebrates; *P*<0.0001, Figure 6b), implying that all codon positions are similarly affected.

In search of additional evidence to support that host-induced substitution (and not translational selection) is the major driving force leading to CpT(ApG) depletion and CpG excess among large DNA viruses infecting invertebrates we sought to investigate the difference between genome-wide dinucleotide O/E ratios and dinucleotide O/E ratios in the coding DNA sequence for a given dinucleotide. If CpT depletion is primarily driven by pressures other than translational selection (eg. mutational pressure), one would expect that the genome-wide CpT_O/E_ ratio will be lower than the CDS CpT_O/E-CDS_ ratio. On the contrary, if translational selection were the major driving force for CpT depletion, one would expect that the depletion of CDS (CpT_O/E-CDS_) ratio will be more pronounced than the depletion of genome-wide CpT_O/E_ ratio. Among viruses infecting invertebrates, the genome-wide depletion of CpT dinucleotides was more pronounced as compared to that within the CDS (*P* = 0.002; Wilcoxon signed rank test) (Table 1). Similarly, the genome-wide gain in CpG dinucleotides was more pronounced as compared to that within the CDS (*P*<0.0001; Wilcoxon signed rank test) (Table 1).

**Table 1:** CpT and CpG dinucleotide frequencies: Genome-wide vs coding DNA sequences (CDS) among large DNA viruses infecting invertebrates.

	Genome-wideO/E ratio	CDS O/Eratio	Viruses with increased bias in the non-codingregion than in coding region (n)	Wilcoxon signedrank test
CpT dinucleotide	0.72±0.10	0.73±0.11	57	P = 0.002
CpG dinucleotide	1.41±0.29	1.39±0.28	73	P<0.0001

**Figure 7:**
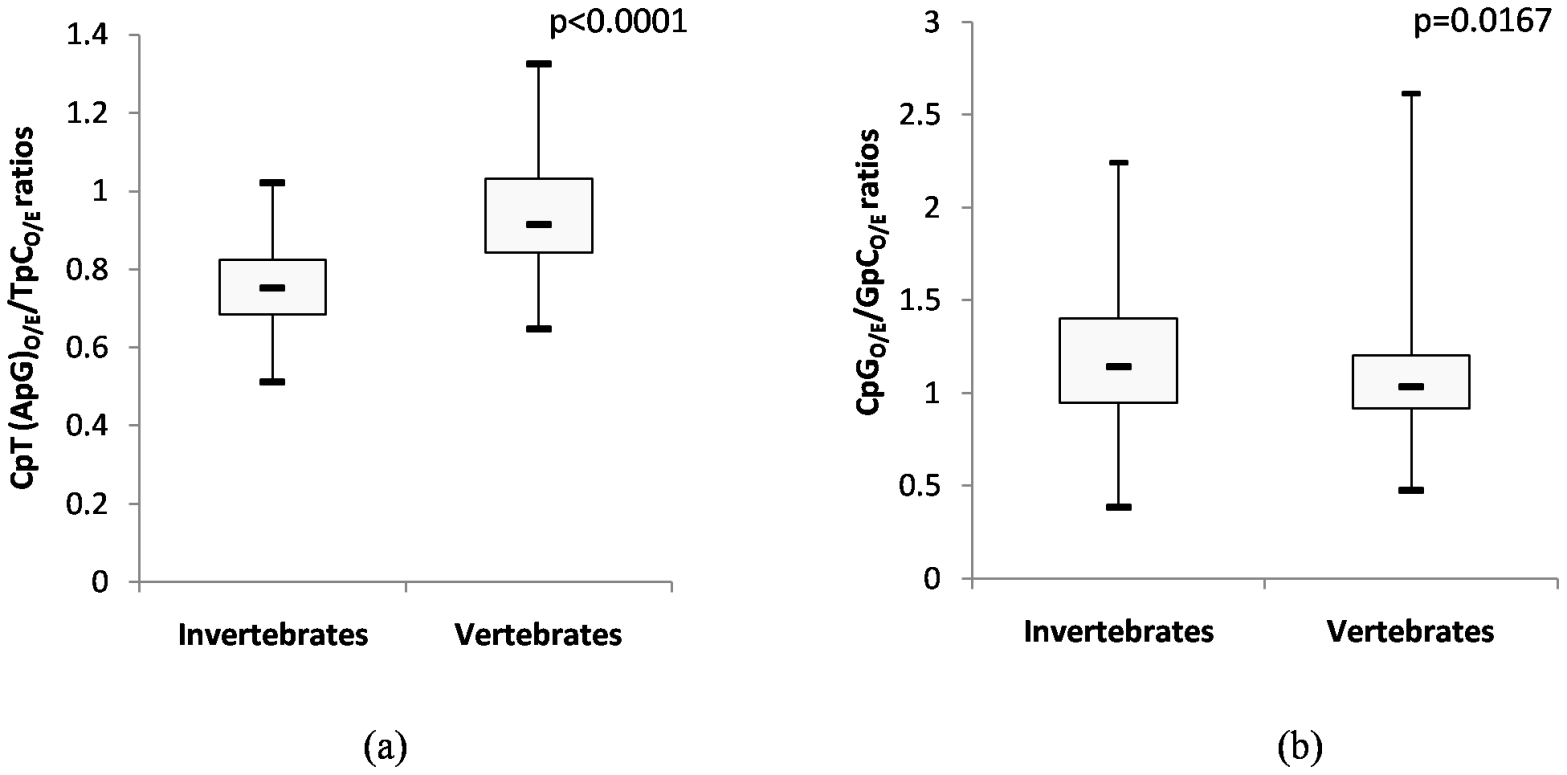
Invertebrate host-induced pressure is specific to CpT (and not TpC) and CpG (and not GpC) dinucleotides. (a) Box plot comparing the CpT_O/E_/TpC_O/E_ ratios among large DNA viruses infecting invertebrates and vertebrates. The CpT_O/E_/TpC_O/E_ ratios were significantly lower in large DNA viruses infecting invertebrates as compared to those infecting vertebrates (0.76±0.11 vs 0.93±0.14; *P*<0.0001) clearly demonstrating that CpT dinucleotides but not TpC dinucleotides are subjected to host-induced pressures. (b) Box plot comparing the relative CpG_O/E_/GpC_O/E_ ratios dinucleotides among large DNA viruses infecting invertebrates and vertebrates. The CpG_O/E_/GpC_O/E_ ratios among large DNA viruses infecting invertebrates were significantly higher than those infecting vertebrates (1.17±0.32 vs 1.06±0.28; *P* = 0.01), demonstrating that CpG dinucleotides but not GpC dinucleotides are subjected to host-induced pressures among large DNA viruses infecting invertebrate hosts.

The CpT dinucleotide is amenable to methylation, while the TpC dinucleotide is not. We investigated the CpT_O/E_/TpC_O/E_ ratios for the viruses studied. The CpT_O/E_/TpC_O/E_ ratios were significantly lower in large DNA viruses infecting invertebrates as compared to those infecting vertebrates (0.76±0.11 vs 0.93±0.14; *P*<0.0001; Figure 7a), clearly demonstrating that CpT dinucleotides but not TpC dinucleotides are amenable to invertebrate host-induced substitutions. Similarly, the CpG_O/E_/GpC_O/E_ ratios among large DNA viruses infecting invertebrates were significantly higher than those infecting vertebrates (1.17±0.32 vs1.06±0.28; *P* = 0.01; Figure 7b).

**Figure 8:**
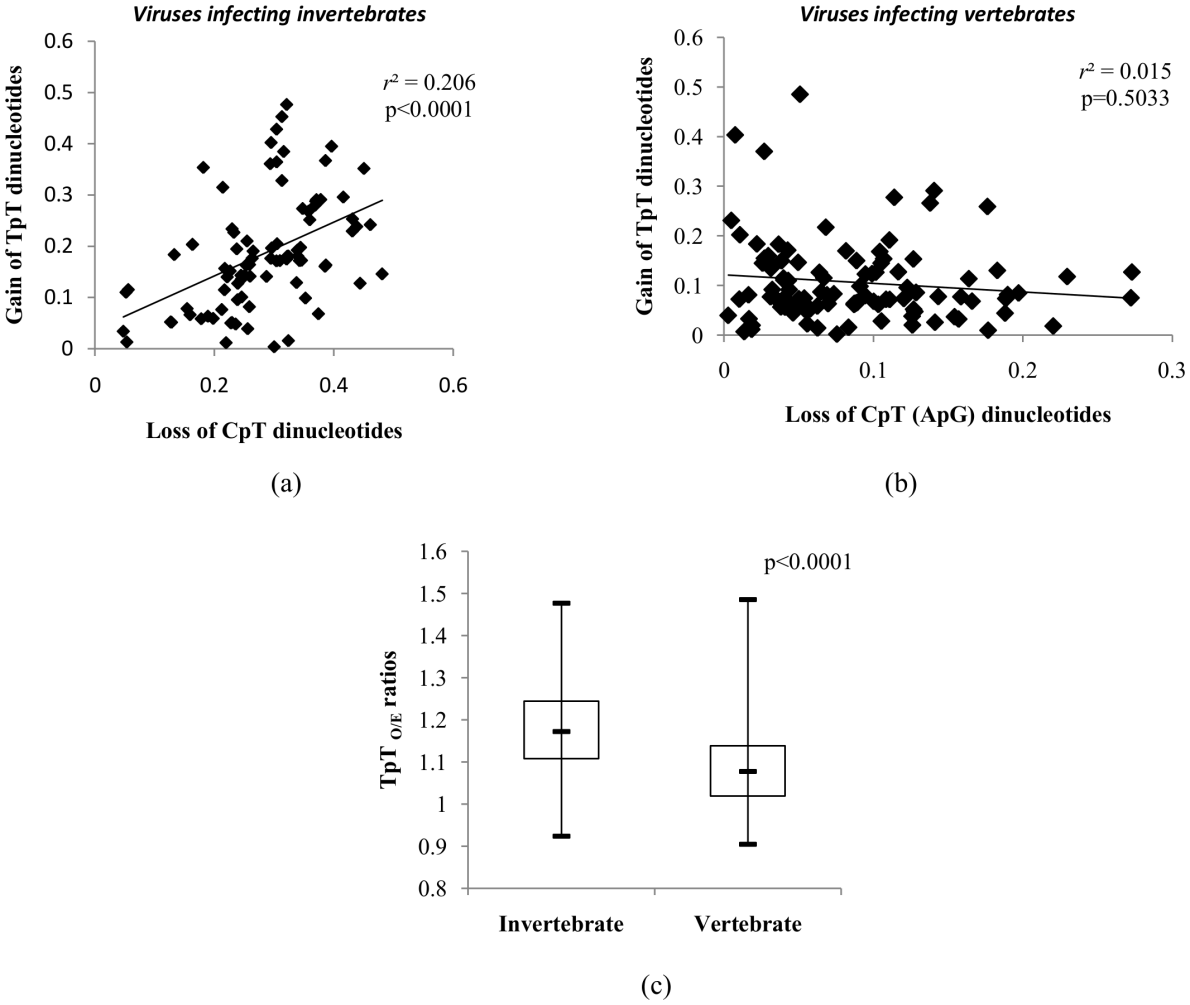
Host methylation capabilitites may be linked to the depletion of CpT dinucleotides among large DNA viruses infecting invertebrates. (a) Scatter plot demonstrating a positive correlation between the loss of CpT dinucleotides (X-axis) and the gain of TpT dinucleotides (Y-axis) in large DNA viruses infecting invertebrate hosts. (b) There was no correlation between the loss of CpT dinucleotides (X-axis) and the gain of TpT dinucleotides (Y-axis) among large DNA viruses infecting vertebrate hosts. (c) Box plot showing the distribution of TpT dinucleotides among large DNA viruses infecting invertebrate and vertebrate hosts. The TpT_O/E_ ratios were significantly higher among the large DNA viruses infecting invertebrates as compared to those infecting vertebrates (mean±SD: 1.17±0.13 vs 1.08±0.11; *P*<0.0001).

Deamination of methylated cytosines results in C to T transitions [12], [13]. The depletion of CpT(ApG) dinucleotides by deamination of 5-methylcytosine within the CpT dinucleotides will lead to a gain of TpT(ApA) dinucleotides. Interestingly, the loss of CpT dinucleotides among large DNA viruses infecting invertebrates correlates to a gain in TpT dinucleotide (Figure 8a; *r* ^2^ = 0.206; *P*<0.0001). There was no correlation between the relative abundance of CpT (ApG) dinucleotides and TpT(ApA) dinucleotides among large DNA viruses infecting vertebrate hosts (Figure 8b; *r* ^2^ = 0.015; *P* = 0.503). In addition, TpT_O/E_ ratios was significantly higher among the large DNA viruses infecting invertebrates as compared to those infecting vertebrates (mean±SD: 1.17±0.13 vs 1.08±0.11; *P*<0.0001) (Figure 8c).

**Figure 9:**
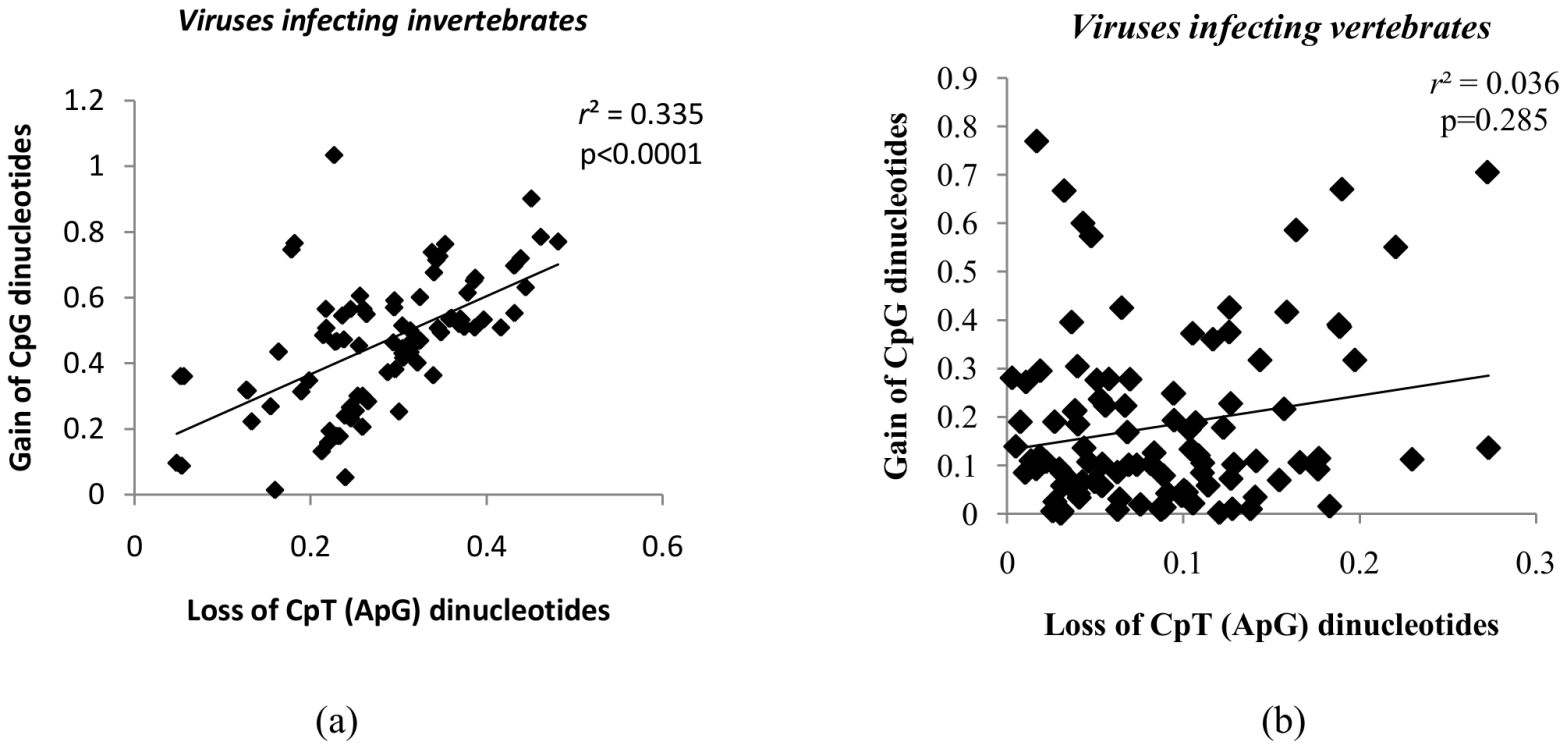
Inverse correlation between CpT loss and CpG gain among large DNA viruses infecting invertebrates. (a) Scatter plot demonstrating a positive correlation between the loss of CpT (ApG) dinucleotides and the gain of CpG dinucleotides (Y-axis) among viruses infecting invertebrate hosts. (b) Scatter plot demonstrating a lack of correlation between the loss of CpT (ApG) dinucleotides and the gain of CpG dinucleotides (Y-axis) among viruses infecting vertebrate hosts.

The loss of CpT(ApG) dinucleotides correlated with a gain in CpG dinucleotides among large DNA viruses infecting invertebrate hosts (Figure 9a; *r^2^* = 0.335; *P*<0.0001). However, there was no correlation between the relative abundance of CpT(ApG) and CpG dinucleotides among the large DNA viruses infecting vertebrate hosts (Figure 9b; *r^2^* = 0.036; *P* = 0.28).

## Discussion

### Systematic CpT(ApG) depletion and CpG excess among large DNA viruses infecting invertebrate hosts

We investigated the relative abundance of dinucleotides among large DNA viruses infecting a wide range of invertebrates and vertebrates hosts. We found systematic CpT(ApG) depletion and CpG excess among large DNA viruses infecting invertebrate hosts (Figure 1a). In contrast, there was no major variation in the relative abundance of CpT and CpG dinucleotides among large DNA viruses infecting vertebrate hosts (Figure 1b). The CpT_O/E_ ratios were significantly lower among the large DNA viruses infecting invertebrates as compared to those infecting vertebrates (Figure 2a; 0.72±0.10vs 0.96±0.09; *P*<0.0001). The CpG_O/E_ ratios were significantly higher among the large DNA viruses infecting invertebrates as compared to those infecting vertebrates (Figure 2b; 1.41±0.29 vs 0.99±0.26; *P*<0.0001). Depletion of TpA dinucleotides was a common feature among both the groups of viruses (Figure 1a and 1b). Avoidance of stop codons (UAG and UAA) and increased susceptibility of UpA to cytoplasmic ribonucleases [27] may explain the depletion of TpA dinucleotides.

The depletion of CpT dinucleotides and the presence of CpG excess appears to be a unique genomic signature of large DNA viruses infecting invertebrate hosts. To the best of our knowledge, neither CpT depletion nor CpG excess have been described among any group of viruses. Intrigued by this finding, we went on to investigate the underlying mechanisms that could potentially contribute to this unique genomic signature of large DNA viruses infecting invertebrates.

### CpG_O/E_ ratios are not influenced by GC content

Several studies have demonstrated a positive correlation between CpG_O/E_ ratios and GC content [25], [26]. In our study, we found no correlation between GC content and the CpG_O/E_ ratios (Figure 3a: *r^2^* = 0.057; *P* = 0.22 for the large DNA viruses infecting invertebrates; and Figure 3b: *r^2^* = 0.017; *P* = 0.18 for large DNA viruses infecting vertebrates). Despite their high CpG_O/E_ ratios, large DNA viruses infecting invertebrates had lower GC content as compared to those infecting vertebrates (mean±SD: 0.41±0.08 vs 0.51±0.13; *P*<0.0001). Thus, it is clear that higher CpG_O/E_ ratios among large DNA viruses infecting invertebrates are not linked to higher GC content.

### Translational pressure/codon usage bias does not shape evolution of large DNA viruses

After having demonstrated CpT depletion and CpG excess among large DNA viruses infecting invertebrates we asked the question if these differences arose because of translational selection or host-induced pressures other than translational selection.

A previous report investigating 41 large DNA viruses infecting vertebrates found no major codon usage bias [11]. In our study, all ENC values were above 40, suggesting the absence of major codon usage biases in the viruses studied. The ENC values for most viruses in both groups were either on the ENC* curve (expected ENC values) or just below it in the ENC-GC_3_ plot (Figure 4a and 4b). This finding also implies that the observed codon usage bias is explained by the underlying differences in nucleotide composition, supporting the role of host-induced pressures other than translational selection.

We then used the ENC′ statistic, which corrects for the influence of uneven base composition [24], [28]. The greater the GC content departs from 0.5, the higher the difference between ENC′ and ENC in both groups of viruses studied (Figure 5a and 5b). Most ENC′ values were closer to 61 (representing no codon usage bias) than were ENC values, implying that the observed differences in codon usage bias are influenced by underlying differences in nucleotide composition. Taken together, the ENC statistic and the ENC′ statistic support (a) the absence of major codon usage biases among the viruses studied and (b) the notion that host-induced pressures other than translational selection shapes the evolution of large DNA viruses infecting vertebrates and invertebrates.

Codon usage bias across different species [29]–[31] and also within different cell types of a given species are well documented [32]. We found no evidence of strong codon usage bias among the viruses we studied. A possible explanation for this may be that low codon usage bias may be beneficial for the virus as it is likely to facilitate efficient replication across multiple cell types of a species or even across different species.

### Host-induced pressures other than translational selection lead to CpT depletion and CpG excess among large DNA viruses infecting invertebrates

To support the notion that host-induced pressures other than translational pressure is the major force contributing to the observed differences in nucleotide composition and codon usage bias comes from analysis of the correlation between GC_3_ and GC_1,2_. If a poor correlation between GC_3_ and GC_1,2_ (reflecting the presence of codon position-dependent differences in nucleotide composition) is observed it suggests a major role for translational pressure; while a good correlation between GC_3_ and GC_1,2_ supports the role of mutational pressure (since all codon positions are similarly affected) in shaping the nucleotide composition of the genome. We found significant correlation between GC_3_ and GC_1,2_ among viruses infecting invertebrate hosts (*r^2^ = *0.943; *P*<0.0001; Figure 6a) and those infecting vertebrate hosts (*r* ^2^ = 0.960; *P*<0.0001; Figure 6b) vindicating the role of host-induced pressures other than translational pressure in shaping the evolution of large DNA viruses. This finding further supports the notion that nucleotide composition of the viruses studied is primarily governed by host-induced pressures other than translational pressure.

Additional evidence linking host-induced pressures other than translational pressure to CpT depletion and CpG excess among large DNA viruses infecting invertebrates comes from analysis of differences between genome-wide O/E ratios and coding region (CDS) O/E ratios for CpT and CpG dinucleotides. The genome-wide depletion of CpT dinucleotides and the genome-wide over-representation of CpG dinucleotides were more pronounced as compared to that with the CDS (Table 1).

Taken together, these findings unambiguously support the role of genome-wide substitutions as the major driving force leading to CpT depletion and CpG excess among large DNA viruses infecting invertebrates. Our finding that genome-wide substitutions dominate translational selection of specific codons is in keeping with previous reports on other viruses [32], [33].

### Host methylation capabilities may be linked to CpT(ApG) depletion and CpG excess

Having demonstrated that host-induced pressures other than translational pressure contribute to CpT(ApG) depletion and CpG excess among large DNA viruses infecting invertebrate hosts we investigated if the TpC dinucleotide is also under a similar pressure. The TpC(GpA) dinucleotide has the same mononucleotide composition (C and T or A and G ) as CpT(ApG) dinucleotides.

The near-normal TpC(GpA)_O/E_ ratios (mean ± SD: 0.96±0.13; *P*<0.0001; Figure 7a) among large DNA viruses infecting invertebrates indicates that the TpC(GpA) dinucleotide is not subjected to similar host-induced substitutions that occur at CpT dinucleotides. In addition, CpT(ApG)_O/E_/TpC(GpA)_O/E_ ratios were significantly lower in large DNA viruses infecting invertebrates host as compared to those infecting vertebrate hosts (0.76±0.11 vs 0.93±0.14; *P*<0.0001) (Figure 7a). This finding reiterates that CpT(ApG) dinucleotides but not TpC(GpA) dinucleotides are subjected to invertebrate host-induced pressures leading to substitutions. In addition, it also suggests that the depletion of CpT dinucleotides in this group of viruses is not linked to general substitutions within the constituent mononucleotides (C and/or T) but to substitutions that are specific to CpT dinucleotides.

Similarly, the CpG_O/E_/GpC_O/E_ ratios among large DNA viruses infecting invertebrates are significantly higher than those infecting vertebrates (Figure 7b, 1.17±0.32 vs 1.06±0.28; *P* = 0.01), suggesting that mechanisms linked to increasing or maintaining CpG dinucleotide content do not influence the GpC dinucleotide content. The CpT (and not TpC) and CpG (and not GpC) dinucleotides of large DNA viruses infecting the invertebrates represent unique targets for substitution, implying that the underlying invertebrate host-induced pressure is likely to be linked to methylation of 5-methylcytosine within the dinucleotides.

Major differences in methylation patterns and in the repertoire of DNA methyltransferases (DNMTs) between vertebrate and invertebrate hosts are well known [5]. Interestingly, non-canonical cytosine methylation in non-CpG dinucleotides, including methylation in CpT dinucleotides has been described among invertebrate hosts [34], [35]. The DNMT2 protein in invertebrates has been linked to CpT and CpA methylation. While DNMT 2 appears to be conserved among vertebrates and invertebrates, the lack of DNA binding domain within invertebrate DNMT2 has been linked to non-canonical cytosine methylation [36]. Given that CpT methylation occurs in invertebrate hosts [35], [37] it is possible that the cytosines within CpT dinucleotides of large DNA viruses infecting invertebrates may also be methylated; subsequent deamination of 5-methylcytosines within CpT dinucleotides will result in a C to T transition leading to the loss of a CpT (ApG) dinucleotide and the gain of a TpT(ApA) dinucleotide. Interestingly, in our study, a significant correlation between the depletion of CpT dinucleotides and the gain in TpT dinucleotides was seen among large DNA viruses infecting invertebrates (Figure 8a; *P*<0.0001); but there was no such correlation among large DNA viruses infecting vertebrate hosts (Figure 8b; *P = *0.503). This finding suggests that deamination of 5-methylcytosine in CpT dinucleotides may, at least in part explain the depletion of CpT dinucleotides among large DNA viruses infecting invertebrates. In addition, higher TpT_O/E_ ratios among the large DNA viruses infecting invertebrates as compared to those infecting vertebrates (mean±SD: 1.17±0.13 vs 1.08±0.11; *P*<0.0001; Figure 8c) strengthens the link between the ability of invertebrates to methylate CpT and the depletion of CpT among large DNA viruses infecting this group of hosts. In addition, this finding argues against random mutations leading to CpT depletion in large DNA viruses infecting invertebrates.

### Correlation between CpT depletion and CpG excess

An earlier study investigating dinucleotide frequencies among completely sequenced vertebrate and invertebrate animal genomes found a correlation between loss of CpG dinucleotides and the gain of CpT dinucleotides [38]. In our study, we demonstrate a correlation between the loss of CpT dinucleotides and the gain of CpG dinucleotides among large DNA viruses infecting invertebrates (Figure 9a; *r^2^* = 0.335; *P*<0.0001); but there was no such correlation among the large DNA viruses infecting vertebrates (Figure 9b; *r^2^* = 0.036; *P* = 0.28). The inverse correlation between the relative abundance of CpT and CpG dinucleotides among large DNA viruses infecting invertebrates is in keeping with finding from earlier studies on animal genomes [38]; however, the reasons for this inverse correlation remain unclear.

### Possible reasons for CpT depletion and CpG excess among DNA viruses infecting invertebrates

Despite major differences in genome organization, replication and host range among DNA viruses infecting invertebrates, CpT depletion and CpG excess have emerged to be the unifying theme across this group of viruses. This finding clearly links host-related factors to CpT depletion and CpG excess. Apart from the potential link between host methylation and the depletion of CpT dinucleotides, our findings do not elucidate specific host-related factors linked to CpT depletion or CpG excess. Two possible explanations are summarized below:

#### (a) CpT dinucleotides are immunostimulatory

The depletion of CpG dinucleotides has been linked to evasion of host immune response via stimulation of Toll-like receptor 9 (TLR9) by unmethylated CpG dinucleotides [19], [39]. TLR9 acts through IL-8 secretion [40] and unmethylated CpG motifs in bacterial DNA induce IL-8 secretion through TLR9 [40], [41]. IL-8 is highly conserved from invertebrates to mammals [42]. Thymidine-rich motifs lacking CpG dinucleotides are immunostimulatory [43]. Importantly, synthetic oligonucleotides containing unmethylated CpT dinucleotides instead of CpG dinucleotides stimulate an interleukin (IL-8) response in human cells [44]. Though merely speculative, we propose that unmethylated CpT dinucleotides may be immunostimulatory among invertebrate hosts as (a) CpT is the only other dinucleotide (apart from CpG dinucleotide) shown to be immunostimulatory (b) induction of IL-8 by both CpG as well as CpT dinucleotides and (c) high frequency of CpT methylation among invertebrates [35], [37], [45]. Our findings do not rule out the possibility that host-induced selection against CpT occurs due to the immunostimulatory nature of unmethylated CpT dinucleotides among invertebrate hosts. It is possible that unmethylated CpT dinucleotides may be linked to pathogen associated molecular patterns among invertebrate hosts.

#### (b) Virus-host co-evolution

The complete genome sequence of most invertebrate and vertebrate hosts of viruses included in our study is currently unavailable. Nonetheless, data from studies analysing a limited number of complete and partial genomes indicate marginal CpT depletion in invertebrate hosts [16], [38]. It is therefore possible that CpT depletion is a common feature of invertebrate genomes and CpT depletion among large DNA viruses infecting invertebrate hosts may reflect virus-host co-evolution.

The absence of TLR9 in invertebrates may potentially allow the maintenance of CpG dinucleotides in invertebrates DNA viruses. A study analysing the CpG content of genes in the *Apis mellifera* (honeybee), a social insect, revealed that genes with a low CpG content (mean CpG_O/E_ = 0.55) were linked to hypermethylation of germline DNA, while those with a high CpG content (mean CpG_O/E_ = 1.5) were linked to hypomethylation of germline DNA [46]. It is therefore possible that lack of CpG methylation among large DNA viruses infecting invertebrates may explain the high CpG content among this group of viruses.

In our study, the presence of excess CpG among large DNA viruses infecting invertebrates suggests that a mechanism to conserve CpGs against depletion of CpGs may exist in this group of hosts. Alternatively, large DNA viruses with increased CpG dinucleotide content may have a survival advantage in invertebrate hosts leading to a positive selection of these strains.

Our findings shed new light on evolutionary differences between large DNA viruses infecting invertebrate hosts and those infecting vertebrate hosts. We have identified depletion of CpT(ApG) dinucleotides and over-representation of CpG dinucleotides as the unique genomic signature for large DNA viruses infecting invertebrates. Our data provides evidence that supports the existence of invertebrate host-induced pressures specifically acting on CpT(ApG) and CpG dinucleotides of the infecting large DNA viruses. We believe that our findings provide a framework to understand invertebrate host-related factors and their role in shaping virus evolution and perhaps virus pathogenesis.

## Supporting Information

Figure S1 **Distribution pattern of CpT and CpG dinucleotides in large DNA viruses.** The distribution pattern of CpT dinucleotides in viruses infecting (a) invertebrates and (b) vertebrates. The distribution pattern of CpG dinucleotides in viruses infecting (a) invertebrates and (b) vertebrates.
(TIF)

Dataset S1 **Accession numbers of virus sequences and host type.**
(XLSX)

## References

[1] Simmonds P, Xia W, Baillie JK, McKinnon K(2013) Modelling mutational and selection pressures on dinucleotides in eukaryotic phyla–selection against CpG and UpA in cytoplasmically expressed RNA and in RNA viruses. BMC Genomics 14: 610 LID- 10.1186/1471-2164-1. 10.1186/1471-2164-14-610 PMC3829696 24020411

[2] Belalov IS, Lukashev AN(2013) Causes and implications of codon usage bias in RNA viruses. PLoS One 8: e56642 LID- 10.1371/journal.pon. 10.1371/journal.pone.0056642 PMC3581513 23451064

[3] Upadhyay M, Samal J, Kandpal M, Vasaikar S, Biswas B et al. (2013) CpG dinucleotide frequencies reveal the role of host methylation capabilities in parvovirus evolution. J Virol 87: 13816-24 LID −10.1128/JVI. 10.1128/JVI.02515-13 PMC3838256 24109231

[4] Sanjuan R, Nebot MR, Chirico N, Mansky LM, Belshaw R (2010) Viral mutation rates. J Virol 84: 9733-48 LID −10.1128/JVI. 10.1128/JVI.00694-10 PMC2937809 20660197

[5] Tweedie S, Charlton J, Clark V, Bird A (1997) Methylation of genomes and genes at the invertebrate-vertebrate boundary. Mol Cell Biol 17: 1469–1475. 10.1128/mcb.17.3.1469 PMC231872 9032274

[6] Regev A, Lamb MJ, Jablonka E (1998) The role of DNA methylation in invertebrates: developmental regulation or genome defense? Mol. Biol. Evol (15) 880–891.

[7] Hendrich B, Tweedie S (2003) The methyl-CpG binding domain and the evolving role of DNA methylation in animals. Trends Genet 19: 269–277. 10.1016/S0168-9525(03)00080-5 12711219

[8] Bird AP (1980) DNA methylation and the frequency of CpG in animal DNA. Nucleic Acids Res 8: 1499–1504. 10.1093/nar/8.7.1499 PMC324012 6253938

[9] Schorderet DF, Gartler SM (1992) Analysis of CpG suppression in methylated and nonmethylated species. Proc Natl Acad Sci U S A 89: 957–961. 10.1073/pnas.89.3.957 PMC48364 1736311

[10] Hoelzer K, Shackelton LA, Parrish CR (2008) Presence and role of cytosine methylation in DNA viruses of animals. Nucleic Acids Res 36: 2825–37. 10.1093/nar/gkn121 PMC2396429 18367473

[11] Shackelton LA, Parrish CR, Holmes EC (2006) Evolutionary basis of codon usage and nucleotide composition bias in vertebrate DNA viruses. J Mol Evol 62: 551–563. 10.1007/s00239-005-0221-1 16557338

[12] Coulondre C, Miller JH, Farabaugh PJ, Gilbert W (1978) Molecular basis of base substitution hotspots in Escherichia coli. Nature 274: 775–780. 10.1038/274775a0 355893

[13] Wiebauer K, Neddermann P, Hughes M, Jiricny J (1993) The repair of 5-methylcytosine deamination damage. EXS 64: 510–522. 10.1007/978-3-0348-9118-9_23 8380355

[14] Chinnery HR, McLenachan S, Binz N, Sun Y, Forrester JV, et al (2012) TLR9 ligand CpG-ODN applied to the injured mouse cornea elicits retinal inflammation. Am J Pathol 180: 209–20. 22085974 10.1016/j.ajpath.2011.09.041 PMC3338340

[15] Holmes EC (2003) Patterns of intra- and interhost nonsynonymous variation reveal strong purifying selection in dengue virus. J Virol 77: 11296–11298. 10.1128/JVI.77.20.11296-11298.2003 PMC224983 14512579

[16] Lobo FP, Mota BE, Pena SD, Azevedo V, Macedo AM, et al (2009) Virus-host coevolution: common patterns of nucleotide motif usage in Flaviviridae and their hosts. PLoS One 4: e6282 doi:10.1371/journal.pone.0006282 10.1371/journal.pone.0006282 PMC2707012 19617912

[17] Bird A (2002) DNA methylation patterns and epigenetic memory. Genes Dev 16: 6–21. 10.1101/gad.947102 11782440

[18] Greenbaum BD, Levine AJ, Bhanot G, Rabadan R (2008) Patterns of evolution and host gene mimicry in influenza and other RNA viruses. PLoS Pathog 4: e1000079 LID- 10.1371/journal.ppa. 10.1371/journal.ppat.1000079 PMC2390760 18535658

[19] Akira S, Uematsu S, Takeuchi O (2006) Pathogen recognition and innate immunity. Cell 124: 783–801. 16497588 10.1016/j.cell.2006.02.015

[20] Willis DB, Granoff A (1980) Frog virus 3 DNA is heavily methylated at CpG sequences. Virology 107: 250–257. 6255678 10.1016/0042-6822(80)90290-1

[21] Tidona CA, Schnitzler P, Kehm R, Darai G (1996) Identification of the gene encoding the DNA (cytosine-5) methyltransferase of lymphocystis disease virus. Virus Genes 12: 219–229. 10.1007/BF00284642 8883359

[22] Federici BA, Bigot Y (2003) Origin and evolution of polydnaviruses by symbiogenesis of insect DNA viruses in endoparasitic wasps. J Insect Physiol 49: 419–432. 10.1016/s0022-1910(03)00059-3 12770621

[23] Wright F (1990) The effective number of codons' used in a gene. Gene87: 23–29. 10.1016/0378-1119(90)90491-9 2110097

[24] Novembre JA (2002) Accounting for background nucleotide composition when measuring codon usage bias. Mol Biol Evol 19: 1390–1394. 10.1093/oxfordjournals.molbev.a004201 12140252

[25] Bernardi G, Olofsson B, Filipski J, Zerial M, Salinas J, et al (1985) The mosaic genome of warm-blooded vertebrates. Science 228: 953–958. 4001930 10.1126/science.4001930

[26] Jabbari K, Bernardi G (1998) CpG doublets, CpG islands and Alu repeats in long human DNA sequences from different isochore families. Gene 224: 123–127. 10.1016/s0378-1119(98)00474-0 9931467

[27] Beutler E, Gelbart T, Han JH, Koziol JA, Beutler B (1989) Evolution of the genome and the genetic code: selection at the dinucleotide level by methylation and polyribonucleotide cleavage. Proc Natl Acad Sci U S A 86: 192–196. 10.1073/pnas.86.1.192 PMC286430 2463621

[28] Fuglsang A (2006) Accounting for background nucleotide composition when measuring codon usage bias: brilliant idea, difficult in practice. Mol Biol Evol 23: 1345–1347. 10.1093/molbev/msl009 16679346

[29] Sharp PM, Bailes E, Grocock RJ, Peden JF, Sockett RE (2005) Variation in the strength of selected codon usage bias among bacteria. Nucleic Acids Res 33: 1141–1153. 10.1093/nar/gki242 PMC549432 15728743

[30] Botzman M, Margalit H (2011) Variation in global codon usage bias among prokaryotic organisms is associated with their lifestyles. Genome Biol 12: R109. 10.1186/gb-2011-12-10-r109 PMC3333779 22032172

[31] Behura SK, Severson DW (2013) Codon usage bias: causative factors, quantification methods and genome-wide patterns: with emphasis on insect genomes. Biol Rev Camb Philos Soc 88: 49–61. 10.1111/j.1469-185X.2012.00242.x 22889422

[32] Ren L, GaoG, Zhao D, Ding M, Luo J, et al. (2007) Developmental stage related patterns of codon usage and genomic GC content: searching for evolutionary fingerprints with models of stem cell differentiation. Genome Bio l8. 10.1186/gb-2007-8-3-r35 PMC1868930 17349061

[33] Butt AM, Nasrullah I, Tong Y (2014) Genome-wide analysis of codon usage and influencing factors in chikungunya viruses. PLoS One 9: e90905 LID- 10.1371/journal.pon. 10.1371/journal.pone.0090905 PMC3942501 24595095

[34] Feliciello I, Parazajder J, Akrap I, Ugarkovic D (2013) First evidence of DNA methylation in insect Tribolium castaneum: environmental regulation of DNA methylation within heterochromatin. Epigenetics 8: 534–41. 23644818 10.4161/epi.24507 PMC3741223

[35] Kunert N, Marhold J, Stanke J, Stach D, Lyko F (2003) A Dnmt2-like protein mediates DNA methylation in Drosophila. Development 130: 5083–5090. 12944428 10.1242/dev.00716

[36] Federica B, Mauro M (2004) The structure of insect DNA methyltransferase 2 (DNMT2) DNA binding domain is responsible for the non-CpG methylation in insect genomes. Caryologia 57: 305–311.

[37] Suzuki MM, Bird A (2008) DNA methylation landscapes: provocative insights from epigenomics. Nat Rev Genet 9: 465–76. 10.1038/nrg2341 18463664

[38] Simmen MW (2008) Genome-scale relationships between cytosine methylation and dinucleotide abundances in animals. Genomics 92: 33–40. 10.1016/j.ygeno.2008.03.009 18485662

[39] Dorn A, Kippenberger S (2008) Clinical application of CpG-, non-CpG-, and antisense oligodeoxynucleotides as immunomodulators. Curr Opin Mol Ther 10: 10–20. 18228177

[40] Jozsef L, Khreiss T, El Kebir D, Filep JG (2006) Activation of TLR-9 induces IL-8 secretion through peroxynitrite signaling in human neutrophils. J Immunol 176: 1195–1202. 16394009 10.4049/jimmunol.176.2.1195

[41] Parilla NW, Hughes VS, Lierl KM, Wong HR, Page K (2006) CpG DNA modulates interleukin 1beta-induced interleukin-8 expression in human bronchial epithelial (16HBE14o-) cells. Respir Res 7: 84. 10.1186/1465-9921-7-84 PMC1489942 16740161

[42] Ottaviani E, Franchini A, Malagoli D, Genedani S (2000) Immunomodulation by recombinant human interleukin-8 and its signal transduction pathways in invertebrate hemocytes. Cell Mol Life Sci 57: 506–513. 10.1007/PL00000711 PMC11146889 10823250

[43] Vollmer J, Weeratna RD, Jurk M, Samulowitz U, McCluskie MJ, et al (2004) Oligodeoxynucleotides lacking CpG dinucleotides mediate Toll-like receptor 9 dependent T helper type 2 biased immune stimulation. Immunology 113: 212–223. 15379982 10.1111/j.1365-2567.2004.01962.x PMC1782571

[44] Kim D, Jung J, Lee Y, Kwon HJ (2011) Novel immunostimulatory phosphodiester oligodeoxynucleotides with CpT sequences instead of CpG motifs. Mol Immunol 48: 1494–1504. 10.1016/j.molimm.2011.04.009 21529949

[45] Takayama S, Dhahbi J, Roberts A, Mao G, Heo SJ et al. (2014) Genome methylation in D. melanogaster is found at specific short motifs and is independent of DNMT2 activity. Genome Res. [Epub ahead of print]. 10.1101/gr.162412.113 PMC4009611 24558263

[46] Elango N, Hunt BG, Goodisman MA, Yi SV (2009) DNA methylation is widespread and associated with differential gene expression in castes of the honeybee, Apis mellifera. Proc Natl Acad Sci USA 106: 11206–11. 19556545 10.1073/pnas.0900301106 PMC2708677

