# Supplementary figures and images for: Systematic CpT (ApG) Depletion and CpG Excess Are Unique Genomic Signatures of Large DNA Viruses Infecting Invertebrates
Source: PLoS One. 2014 Nov 4;9(11):e111793. doi: 10.1371/journal.pone.0111793 (PMC4219779; doi:10.1371/journal.pone.0111793)

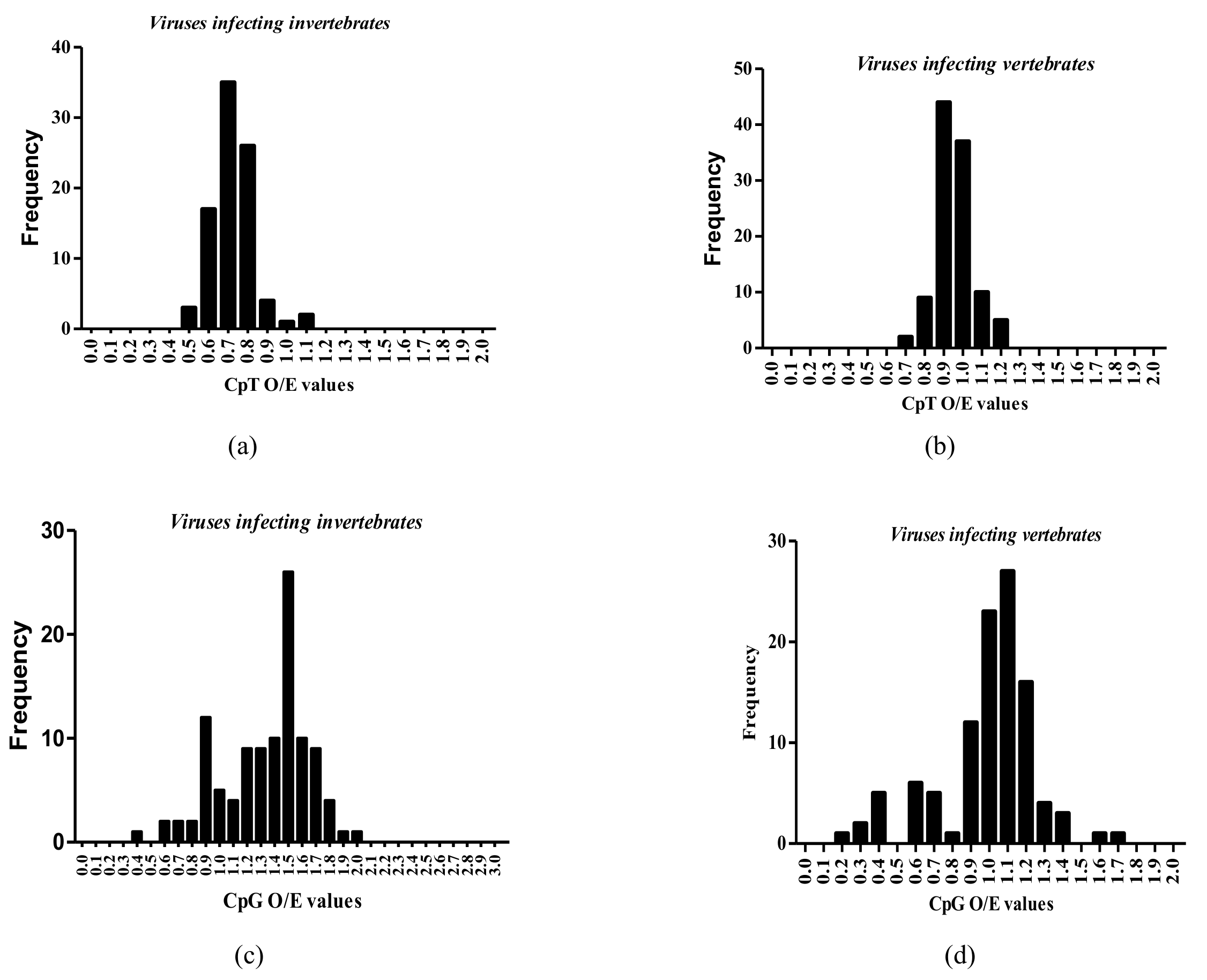

Supplement: Figure S1 — Distribution pattern of CpT and CpG dinucleotides in large DNA viruses. The distribution pattern of CpT dinucleotides in viruses infecting (a) invertebrates and (b) vertebrates. The distribution pattern of CpG dinucleotides in viruses infecting (a) invertebrates and (b) vertebrates. (TIF) [file pone.0111793.s001.tif]
